# Effect of straining on diaphragmatic crura with identification of the straining-crural reflex. The "reflex theory" in gastroesophageal competence

**DOI:** 10.1186/1471-230X-4-24

**Published:** 2004-09-30

**Authors:** Ahmed Shafik, Ali A Shafik, Olfat El Sibai, Randa M Mostafa

**Affiliations:** 1 Department of Surgery and Experimental Research, Faculty of Medicine, Cairo University, Cairo, Egypt; 2 Department of Surgery, Faculty of Medicine, Menoufia University, Shebin El-Kom, Egypt; 3 Department of Physiology, Faculty of Medicine, Banha University, Egypt

## Abstract

**Background:**

The role of the crural diaphragm during increased intra-abdominal pressure is not exactly known. We investigated the hypothesis that the crural diaphragm undergoes reflex phasic contraction on elevation of the intra-abdominal pressure with a resulting increase of the lower esophageal pressure and prevention of gastro-esophageal reflux.

**Methods:**

The esophageal pressure and crural diaphragm electromyographic responses to straining were recorded in 16 subjects (10 men, 6 women, age 36.6 ± 11.2 SD years) during abdominal hernia repair. The electromyogram of crural diaphragm was recorded by needle electrode inserted into the crural diaphragm, and the lower esophageal pressure by a saline-perfused catheter. The study was repeated after crural anesthetization and after crural infiltration with saline.

**Results:**

The crural diaphragm exhibited resting electromyographic activity which showed a significant increase on sudden (coughing, p < 0.001) or slow sustained (p < 0.01) straining with a mean latency of 29.6 ± 4.7 and 31.4 ± 4.5 ms, respectively. Straining led to elevation of the lower esophageal pressure which was coupled with the increased electromyographic activity of the crural diaphragm. The crural response to straining did not occur during crural diaphragm anesthetization, while was not affected by saline infiltration. The lower esophageal pressure declined on crural diaphragm anesthetization.

**Conclusions:**

Straining effected an increase of the electromyographic activity of the crural diaphragm and of the lower esophageal pressure. This effect is suggested to be reflex in nature and to be mediated through the "straining-crural reflex". The crural diaphragm seems to play a role in the lower esophageal competence mechanism. Further studies are required to assess the clinical significance of the current results in gastro-esophageal reflux disease and hiatus hernia.

## Background

Swallowing is a physiologic process by which the food bolus is transmitted from the pharynx to the stomach without esophagopharyngeal or gastro-esophageal reflux [1]. A sphincteric action exists within the lower 4 cm of the esophagus which prevents reflux of gastric contents into the esophagus [2,3]. The mechanism of gastro-esophageal competence is complex and incompletely understood [4-7]. A true anatomical sphincter could not be demonstrated at the lower end of the esophagus, and the sphincter is considered a physiological one [8-11]. The resting pressure within the lower esophageal sphincter (LES) normally exceeds the intragastric pressure by 15–25 cm H_2_O due to tonic contraction of the esophageal musculature [10]. The LES squeeze increases by gastrin and decreases by cholecystokinin, secretin, and glucagons [5,6]. Cholinergic and ∝ – adrenergic stimuli enhance while β – adrenergic stimuli inhibit sphincter contraction^11^. The LES contributes to the prevention of gastric reflux into the esophagus [2,3]; however, the mechanism of action is not exactly known [2-6].

The diaphragm is believed to play a contributory role in the barrier function of the lower esophagus. This auxiliary function seems to be carried out by the crural and not the costal diaphragm. The latter contracts and relaxes with respiration. Crural diaphragm (CD) contraction effects LES pressure increase which is directly proportional to the depth of inspiration at the force of diaphragmatic contraction [12]. Pressure gradients across the esophagogastric junction during expiration is counteracted by the smooth muscle relaxation of the LES, and increases in the gastrocrural pressure gradient caused by the skeletal muscle activity of the diaphragm and abdominal wall are counteracted by the CD [13]. Crural diaphragm has been demonstrated to contribute actively in the process of deglutition [14]. Thus, on crucial balloon distension the CD relaxed, while gastric distension effected CD contraction [14]; this sphincter-like CD action was found to be mediated through the esophago-crural inhibitory and the gastro-esophageal excitatory reflexes, respectively [14].

The role of the CD during increased intra-abdominal pressure is not completely understood. We hypothesized that the CD, upon increase in intra-abdominal pressure by coughing, sneezing or straining, undergoes reflex phasic contraction with a resulting augmentation of the lower esophageal pressure and inhibition of stress reflux of the gastric contents into the esophagus. This hypothesis was investigated in the current communication.

## Methods

### Subjects

Sixteen subjects were enrolled in the study. Ten were men and six women with a mean age of 36.6 ± 11.2 SD years, (range 27–43). The tests were performed during operative repair of an upper abdominal ventral hernia in 9 patients and of incisional hernia after cholecystectomy for calculous cholecystitis in 7 patients. The patients did not complain of swallowing problems in the past or at the time of enrollment. They gave an informed consent after having been fully informed about the nature of the tests to be done and their role in the study.

Physical examination results, including neurologic assessment, were normal. Also barium swallow studies and upper gut endoscopy yielded normal findings. The results of laboratory work including blood count, renal and hepatic function tests as well as electrocardiography were unremarkable.

The study was approved by the Review Board and Ethics Committee of the Cairo University Faculty of Medicine.

### Methods

The EMG activity of the CD was recorded during coughing and during straining. The subjects had received general anesthesia using 5% halothane/ 95% oxygen for their above mentioned hernia operations.

#### EMG activity of the CD

A concentric electromyographic needle electrode of 40 mm in length and 0.65 mm in diameter (Type 13 L 49 Disa, Copenhagen) was introduced into the CD as it encircled the lower end of the esophagus. A ground electrode was applied to the thigh.

A standard electromyographic (EMG) apparatus (Type MES, Medelic, Woking, UK) was used to amplify and display the potentials recorded. Films of the potentials were taken on light-sensitive paper (Linagraph type 1895, Kodak, London, UK) from which measurements of the motor unit action potentials' duration were obtained. The electromyopraphic signals were also stored on an FM tape recorder (type 7758 A, Hewlett-Packard, Waltham, MA) for further analysis as required.

Before performing the experiment, the normality of the EMG activity of the CD was tested by stimulating it with a needle electrode introduced into the CD and registering the motor unit action potentials from the already inserted needle electrode. The CD had normal EMG activity in all examined subjects.

#### Manometric studies

A manometric 6-F catheter was introduced into the esophagus to lie in the high pressure zone at its lower end. The catheter with 2 side ports and a metallic clip applied to its distal closed end for fluoroscopic control was connected to a pneumohydraulic capillary infusion system (Arndorfer Medical Specialities, Greendale, Wis). The pump delivered saline solution continuously via the capillary tube at a rate of 0.6 ml / min. The transducer outputs were registered on a rectilinear recorder (model RS-3400, Gould Inc). Occlusion of the recording orifice produced a pressure elevation rate that was greater than 250 cm H_2_O/s. During pressure measurements, the catheter was rotated so as to record anteroposterior and lateral pressures.

#### Induction of cough and straining

Near the end of the operation when the effect of muscle relaxant had waned, the anesthetist was asked to induce coughing and straining via laryngeal and tracheal stimulation by moving the endotracheal tube while lying in the trachea. The EMG response of the CD to increased intra-abdominal pressure was registered. Readings were recorded during two types of straining: the sudden forcible straining as that induced by coughing, and the slow sustained straining which simulates that occurring during defecation or micturition. The latency of the crural response was measured from the stimulus (straining) to the first deflection of the muscle action potential complex. The millisecond latencies were calculated when the movement artifact associated with straining appeared on the crural EMG and then the time to the first muscle action potential was measured as an index of latency.

#### Crural anesthetization

To define whether the effect of coughing or straining on the crural diaphragm was direct or reflex action, the following lest was done. In 8 subjects (5 men and 3 women), the CD was infiltrated with 5 ml of 2% lidocaine to anesthetize the crura around the needle electrode. The crural response to sudden and slow sustained straining was recorded after 10 minutes and after 2 hours when the anesthetic effect had waned. Similarly, normal saline was injected and the crural response to straining was registered.

The results were analyzed statistically using the Student's t test and values were given as the mean ± standard deviation. Differences assumed significance at p < 0.05.

## Results

**Figure 1:**
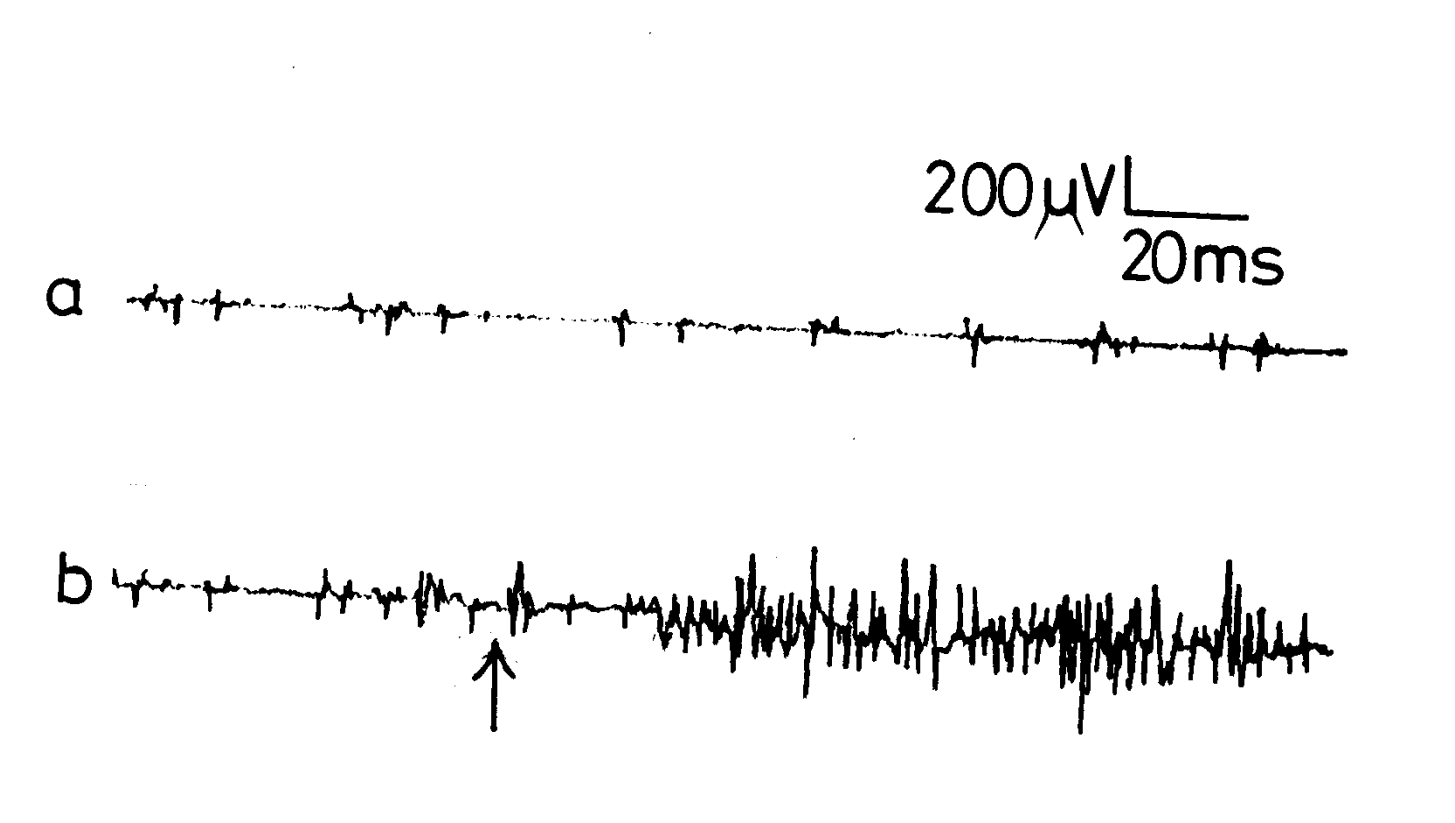
Electromyographic activity of the crural diaphragm a) at rest and b) on sudden straining (coughing). ↑ = coughing

**Figure 2:**
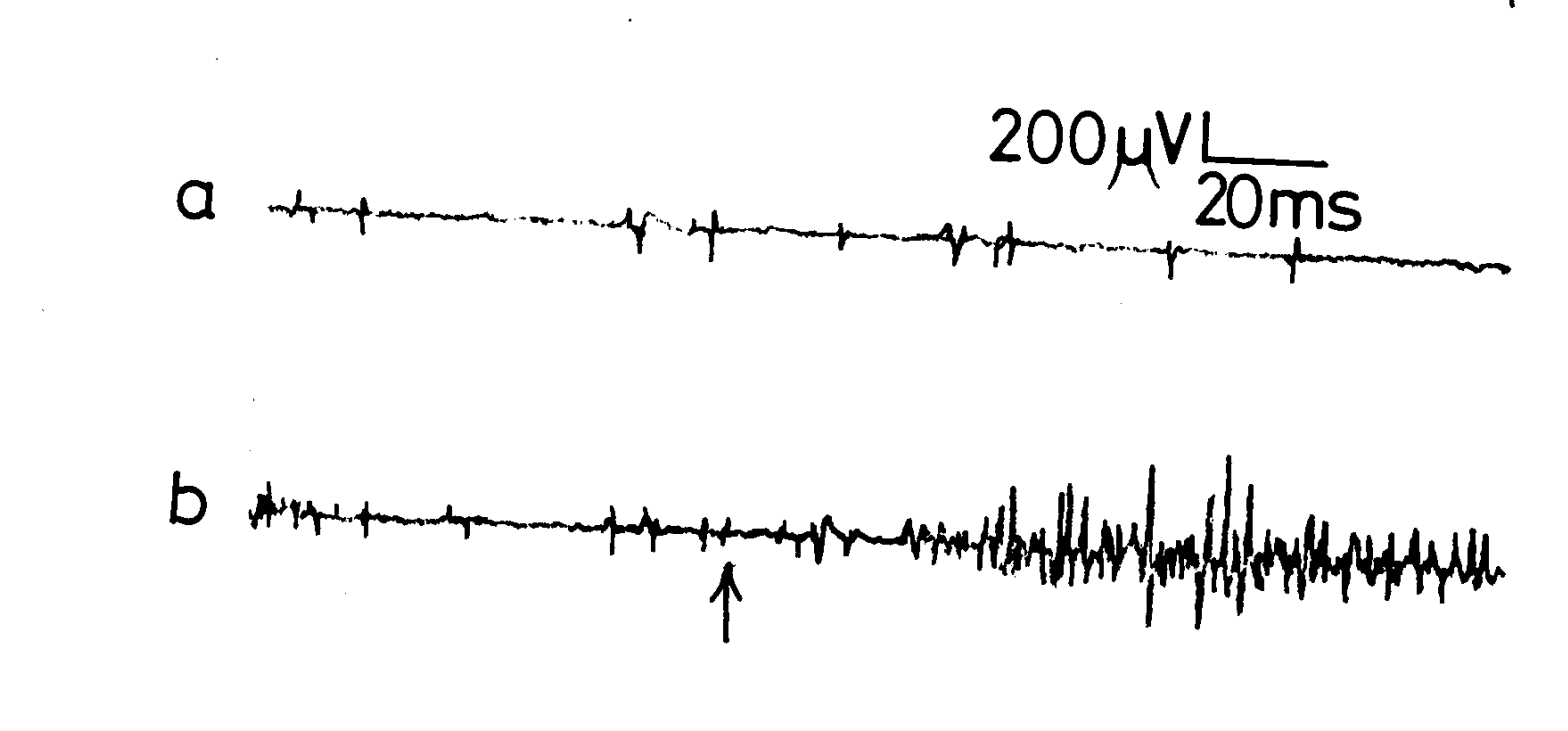
Electromyographic activity of the crural diaphragm a) at rest and b) on slow sustained straining. ↑ = straining

The CD in all of the subjects showed a basal activity with a mean of 112.3 ± 16.3 μV (range 86–123, fig 1). Upon sudden straining (coughing), the CD exhibited an increase in the EMG activity to a mean of 553.6 ± 54.2 μV (range 480–675 μV, p < 0.001, fig 1). The basal activity was resumed after cessation of straining. Slow sustained straining induced increase of the crural EMG activity to a mean of 482.7 ± 42.5 μV (range 366–610, p < 0.01, fig 2).

The crural response to straining (sudden or slow sustained) was reproducible in all studied subjects. It was weaker in women than men, and in the elderly than in the young subjects, though the difference was insignificant (p > 0.05). The CD response disappeared when straining was sustained for more than 15–18 seconds (mean 16.8 ± 1.2) and was not evoked after frequent successive straining. The latency of the response recorded a mean of 29.6 ± 4.7 ms (range 21–33, fig 1) for the sudden straining (fig 1) and 31.4 ± 4.5 ms (range 22–36) for the slow sustained straining (fig 2) with no significant difference between the 2 latencies.

In the 8 subjects in whom the CD was anesthetized, the crural response to straining did not occur, except after 2 hours when the effect of lidocaine had waned; the response after 2 hours was similar to that before anesthetization with no significant difference (p > 0.05). Saline injection of the crura did not affect the crural response to straining.

### Lower esophageal pressure response to straining

The pressure at rest in the LES recorded a mean of 25.4 ± 6.3 cm H_2_O (table 1). On sudden straining (coughing), we registered a mean of 96.6 ± 10.8 cm H_2_O (table 1), while with slow sustained straining a mean of 82.6 ± 8.3 cm H_2_O (table 1). The elevated esophageal pressure was coupled with the increased EMG activity of the CD and was sustained along with the increased motor unit action potentials.

**Table 1:** The pressure in the lower esophageal sphincter at rest and on straining^+^.

	**Pressure (cm H_2_O)**	
	**Mean**	**Range**
Basal	25.4 ± 6.3	17 – 32
Sudden straining	96.6 ± 10.8 *	72 – 124
Sustained straining	82.6 ± 8.3 *	58 – 97

+ values were given as the mean ± standard deviation

* p < 0.01

P values were compared to the basal value.

On CD anesthetization, the lower esophageal pressure dropped to a mean of 14.2 ± 2.4 cm H_2_O (table 1). It rose significantly (p > 0.01) to a mean of 63.7 ± 10.4 cm H_2_O on sudden straining and to a mean of 56.2 ± 7.5 cm H_2_O (p < 0.01, table 2) on slow sustained straining. The pressure returned to the pre-anesthetic level after 2 hours when the anesthetic effect had worn off.

**Table 2:** The pressure in the lower esophagus upon crural anesthetization at rest and on straining^+^.

	**Pressure (cm H_2_O)**	
	**Mean**	**Range**
Basal	14.2 ± 2.4	9 – 18
Sudden straining	63.7 ± 10.4 *	49 – 84
Sustained straining	56.2 ± 7.5 *	43 – 73

+ values were given as the mean ± standard deviation

* p < 0.01

P values were compared to the basal values.

## Discussion

The current study seems to shed some light on the effect of coughing-or-straining-induced intra-abdominal pressure increase on the CD and the lower esophagus. The CD has a respiratory rhythm but is not a respiratory muscle. It surrounds the lower end of the esophagus, which is an intra-abdominal structure and is continuously exposed to variations in the intra-abdominal pressure. The lower esophagus contains a physiologic sphincter, which is the LES. In contrast to the CD which consists of striated muscle fibers, the LES is composed of smooth fibers.

The resting electric activity exhibited by the CD most likely denotes that the CD possesses a resting tone which presumably shares in inducing the high pressure within the LES. The high pressure zone in the lower esophagus appears to be created not only by the effect of the LES but also by the muscle tone of the CD. This is evidenced by the reduced lower esophageal pressure on the CD anesthetization. The increased crural electric activity and the elevated esophageal pressure upon straining presumably denote crural contraction. The CD tone at rest and crural contraction on straining probably share in preventing gastro-esophageal reflux under resting and stress conditions. The disappearance of the crural response on prolonged straining and the non-response after frequent successive straining appear to be due to the fact that the CD consists of striated muscle fibers which are easily fatigable and cannot remain contracted for long periods.

On CD anesthetization, the lower esophageal pressure dropped from the mean basal pressure of 25.4 ± 6.3 cm H_2_O to 14.2 ± 2.4 cm H_2_O. This denotes that the CD has a share of approximately 44% in the basal lower esophageal pressure against 54 % of the lower esophageal sphincter. On straining while the CD was anesthetized, the lower esophageal pressure recorded values significantly below those before anesthetization. These findings would indicate that the CD shares the formation of the lower esophageal high pressure zone with the LES.

The question that needs to be discussed is whether the crural response to straining is the result of a direct action or reflex in nature.

### The straining-crural reflex

The current study have demonstrated that the CD contracts on straining as evidenced by increase of both the crural EMG activity and the lower esophageal pressure. The crural contraction on straining could be a direct or reflex action; it seems to be reflex in nature as became evident from its absence when the CD, a suggested arm of the reflex arc was anesthetized. This reflex relationship was reproducible and we call it the "straining – crural reflex". Lidocaine blocks the sensory fibers (C and A delta – fibers) which are responsible for pain and reflex activity [15,16]. The straining-crural reflex appears to be evoked in conditions of increased intra-abdominal pressure as occurs during coughing, squeezing and during straining at defecation or micturition.

### Role of the straining-crural reflex in lower esophageal competence: The "reflex theory", a new concept

The mechanism of gastroesopageal competence is vague and incompletely understood [2-7]. There are several factors claimed to maintain the lower esophageal competence. These include the "diaphragmatic pinchcock", a circular anatomic sphincter and a flap valve [17,18]. However, in spite of the general acceptance that the circular fibers at the lower esophagus acts as a sphincter, there is so far no anatomical evidence to support the presence of a true sphincter [17-21].

Meanwhile, it is highly probable in the light of the findings of our study that the prevention of gastro-esophageal reflux is a "reflex process" rather than an anatomical entity. We have previously demonstrated that gastric distension by food or an increase in the intra-abdominal pressure would evoke the "gastroesophageal reflex" which acts to tighten the LES [22]. The more voluminous the gastric distension or the higher the intra-abdominal pressure, the tighter the LES.

The current study presumably denotes that the CD shares reflexly in the competence mechanism of the gastroesophageal junction. Thus, upon increase of the intra-abdominal pressure, the straining-crural reflex seems to be evoked effecting crural contraction and increase of the lower esophageal pressure.

In view of the aforementioned results and discussion, we believe that the "reflex theory" plays a more important role in gastroesophageal competence than the diaphragm pinchock, the flap valve mechanism or other possible anatomical factors.

## Conclusion

The CD appears to play a role in the lower esophageal competent mechanism. Straining effected an increase in the EMG activity of the CD and in the lower esophageal pressure. This effect is suggested to be reflex in nature and to be mediated through the "straining-crural reflex". Further studies are needed to evaluate the clinical significance of the current results in the pathogenesis and treatment of gastresophageal disease and hiatus hernia.

## List of abbreviations

lower esophageal sphincter (LES)

crural diaphragm (CD)

electromyographic (EMG)

## Competing interests

The author(s) declare that they have no competing interests.

## Pre-publication history

The pre-publication history for this paper can be accessed here:


